# Assessment of spatiotemporal characteristics of gait, trough the Phyphox® app: a case series

**DOI:** 10.1186/s13256-023-04296-z

**Published:** 2023-12-29

**Authors:** Carolina A. Cabo, Orlando Fernandes, Sara Santos, Jose A. Parraca

**Affiliations:** 1 Departamento de Desporto e Saúde, Escola de Saúde e Desenvolvimento Humano, Universidade de Évora, Largo Dos Colegiais 2, 7000-645 Évora, Portugal; 2 Comprehensive Health Research Centre (CHRC), University of Évora, Largo Dos Colegiais 2, 7000-645 Évora, Portugal; 3 Instituto Politécnico de Setúbal, Escola Superior de Educação, 2914-504 Setúbal, Portugal

**Keywords:** Biomechanics, Case report, Fractals, Gait, Smartphone

## Abstract

**Background:**

Spatiotemporal characteristics from human locomotion can provide effective clinical metrics to assess motor control and brain function. This case report aims to assess the temporal structure of variability in stride-to-stride time and calculated the intrinsic fractal frame that is hidden below the repetitive structure of physiological gait through the “Phyphox” app. This is an innovative study from the perspective of analyzing gait variables through a mobile app.

**Case presentation:**

Five older adults Caucasian (3 women; age = 73 ± 10,5 years; body mass = 62,2 ± 15,1 kg; height = 1,56 ± 0,1 m; 2 men; age = 75,5 ± 7,8 years; body mass = 86,3 ± 18,0 kg; height = 1,77 ± 0,1 m) participated in this study. Five participants were asked to walk with a natural cadence, two of the participants presented a value greater than 80 step’s/minute (81.14 ± 0.01; 86.67 ± 0.02); and the others had values between 55 and 65 step’s/minute (55.20 ± 0.02; 55.78 ± 0.05; 61.02 ± 0.05). Regarding the coefficient of variation, only one participant presented 10.08%. For the total number of steps, three of the participants had values greater than 1000 steps. The variability of these stride-to-stride time has been quantified through detrended fluctuation analysis; one participant presented a value above 1.

**Conclusions:**

This study provides evidence that a smartphone might provide a valid measure to assess the spatiotemporal characteristics of gait.

## Background

Aging is defined as a natural and physiological process that progressively accompanies the life cycle and involves complications in the psychosocial and physical spheres, thus generating slow thinking, depression, functional disability, loss of resistance, inactivity, and physical degeneration. The changes in the rhythmically of the stride-to-stride are important to assesses the motor control and is been demonstrated an increase in variability in stride time intervals between elderly population and healthy adults [13]. Research over the last few decades has examined the properties of adaptive and functional biological systems through gait analysis during treadmill and floor locomotion, showing that healthy and clinical populations can exhibit similar variability in their rhythms despite having different functional behaviours. All biological rhythms exhibit some level of variability, and while some of these systems remain adaptive and functional, others they are maladaptive and dysfunctional. Consequently, the risk of injury increases if the person is not able to adapt their gait [3].

Active aging contributes to the maintenance of functional capacity, quality of life and independence. The functional changes inherent to the aging process can lead to situations of imbalance and, consequently, increase the risk of falling [10].

Worldwide, falls are the second leading cause of accidental death, with those over the age of 65 experiencing the highest proportion of fatal falls. Injury resulting from falls is associated with reduced physical functioning, loss of independence, and fear of future falls, which in turn can lead to reductions in physical activity and social engagement [7].

It becomes relevant to develop preventive measures relating the risk factors for the occurrence of falls. The clinical approach should contain a good history related to past falls and, in addition, the assessment of muscle strength and range of motion as well as performing gait and balance tests. The application of functional tests is intended to assist the clinical evaluation by providing data on the patient’s mobility capacity and revealing possible balance deficits [10].

By providing seniors with appropriate health apps, they can independently and self-determinedly monitor and manage their health. Smartphones have many sensors capable of measuring and tracking vital parameters as well as other health-related data. Healthcare applications analyse and process this data and therefore can provide comprehensive support to healthcare services in the future [8].

More specifically, the rhythmic variability inherent to these systems also exhibited fractal scaling (that is, patterns of variability at one time scale are like those found at other time scales). Thus, more recently, metrics that index the structure of variability have gained favour in the literature because of their ability to quantify the dynamic, time-evolving nature of the locomotor system’s rhythmic behaviour [9].

Technological advances allow the updating of techniques and procedures used by health professionals, such as the use of mobile devices as support devices. Smartphones have great potential as they are accessible, practical, and portable devices, which can help from data collection procedures to the diagnosis of diseases [10, 12].

Within the free license applications available for the Android and IOS operating system, Phyphox [12] has stood out for allowing the use of a variety of sensors to perform various activities, while most applications have its limited use. With interactive graphic resources and real-time measurements, the data obtained is analysed directly in the application, which allows a quick reading even without the use of a computer. The application also offers data export and sharing options, so that they can be processed later and in a more robust program [5].

The Phyphox application was developed at the second Institute of Physics of the Technical University of Aachen, Germany. With this application we can carry out Physics experiments using the smartphone. This is possible because the application allows using the device’s sensors to perform evaluations such as detecting the frequency of a simple pendulum using the smartphone’s own accelerometer sensor. This application can be used free of charge and can be found for download on its official website (phyphox.org). The basic prerequisites to be able to use the application are some sensors, among them: accelerometer, microphone, light sensor, and proximity sensor. In general, the more sensors the device has, the more experiences can be used, as the greater the scope that can be reached [6].

Phyphox is an application available for Android and IOS. It is possible to use the various sensors existing in smartphones with this application, as it has several functions such as: accessing measurements of acceleration, rotation rate and smartphone location. It is also possible to carry out measurements of magnetic field, pressure, light intensity, and sound intensity [11]. By the application it is still possible to obtain a direct estimate of the gravitational acceleration, performed in real time [5].

## Case presentation

### Demographic details

A study was carried out to collect data through a practical test. Data was collected between December 2021 and July 2022 through the recruitment of participants who were members of the physical activities group of the Municipality of Almada (MA), more specifically a project called Active Retirement that consisted of promoting the practice of physical activity with stimulation of the sensorimotor abilities of pre-retirees and retirees, protocol between the University of Évora (UE) and the MA.

From the group of participants, we randomly chose five individuals for this pilot study.

Five older adults (3 women; age = 73 ± 10,5 years; body mass = 62,2 ± 15,1 kg; height = 1,56 ± 0,1 m; 2 men; age = 75,5 ± 7,8 years; body mass = 86,3 ± 18,0 kg; height = 1,77 ± 0,1 m) participated in this study. The study was approved by the Ethics Committee of the University of Évora (approval number: 21040). Each participant provided informed consent prior to participation according to Helsinki for human studies. Prior to any walk test, participants underwent a general health assessment for inclusion criteria and demographics.

### Medical history

Participants under 55 years of age were excluded. The inclusion criteria defined were: (1) age over 65 years; (2) people without dentures (except dental prosthesis); (3) people who have not been operated on for less than 6 months; (4) residents in the municipality of Almada. Exclusion Criteria: (1) people with musculoskeletal diseases; (2) people with locomotion problems; (3) psychiatric diseases and neurological disorders; (4) people with a clinical cardiovascular and cardiopathies.

Initially, an anthropometric and body composition assessment was performed, through weight and height analysis. For 5 participants, 3 female and 2 males, we calculated the BMI index using the Centers for Disease Control and Prevention (CDC) adult calculator.

### Treatment or intervention

We asked participants to complete a 12-minute walk without stimulation (self-paced walking) and were instructed to walk at their self-selected pace while looking straight ahead.

The first 15 seconds were discarded to avoid any initial adaptation. Likewise, the last 15 seconds have been discarded to avoid cadence slowing effects. The mean and coefficient of variation were calculated for each time series of inter-stride intervals (ISIs). The fractal scale exponent, α, was also calculated from the time series of ISIs. The fractal-scaling exponent, α, was also calculated from the ISIs time series (α-ISIs) using DFA.

The log F(n) is plotted against log n (the root mean square versus window sizes). The slope of this graph is the reported α value. If the α value is greater than 0.5, the long-range correlation is positively persistent. This means that increases are likely to be followed by increases and decreases are likely to be followed by decreases. Whereas if the α value is less than 0.5, the long-range correlation is anti-persistent, meaning that increases are likely to be followed by decreases and vice versa. If the α value is greater than 1, the signal is considered brown [1].

In the present study, the stimulus was designed to have an α value of 1, indicating pink noise and fractal complexity in the signal. On the other hand, a lower value indicates loss of complexity. For example, a white noise signal type (that is, highly variable and unstructured) typically has an α value of 0.5. The Detrended Fluctuation Analysis (DFA) window size range selected in the current study was from 16 to N/8, where N is the number of pass intervals.

Nonlinear methods were analyzed through number of steps per min, standard variation, coefficient of variation, total number of steps, and the fractal dimension using the trended fluctuation analysis, from five time series of 10 minute’ walk.

## Results

Five subjects were evaluated, 3 women and 2 men, aged between 60 and 85 years old. According to BMI, we can see that only one of the participants has a normal value, the rest are overweight (Table 1).

**Table 1:** Demographics and sample's characteristics

Participant (code)	Age	Weight	Height	BMI
´009	81	73,6	1,72	24,9
´012	84	69,8	1,57	28,3
´106	70	99	1,83	29,6
´111	72	44,8	1,42	22,2
´113	63	71,9	1,69	25,2

**Fig. 1:**
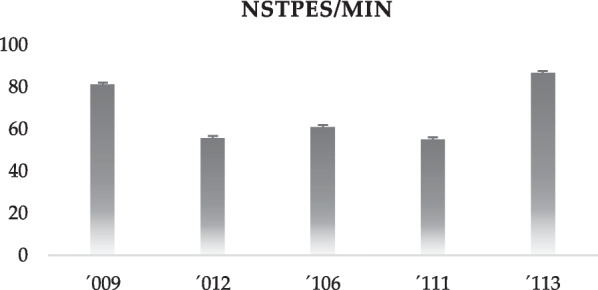
The total number of steps per minute for each participant

Regarding the number of steps per min, two of the participants presented a value greater than eighty steps per min (81,14 ± 0,01; 86,67 ± 0,02); and the others had values between 55 and 65 steps per min (55,20 ± 0,02; 55,78 ± 0,05; 61,02 ± 0,05) (Fig. 1).

Regarding the coefficient of variation, only one participant presented 10.08%, and the others presenting values between 2 and 4%.

The total number of steps, three of the participants had value greater than 1000 steps and the other two approximately 600 steps.

Finally, regarding the fractal dimension, only one participant presented a value above 1, with the remaining values between 0.7 and 0.8 (Table 2).

**Table 2:** Summary results for all the studied gait variables in all conditions

nStpes/min	±	sd	%CV	nSteps	Fractal
81,147	±	0,012	2,688	1309	0,712
55,783	±	0,045	4,851	667	1,059
61,028	±	0,051	10,076	1154	0,685
55,203	±	0,019	2,075	646	0,831
86,678	±	0,016	3,282	1188	0,771

## Discussion and conclusions

This study aimed to assess the temporal structure of variability in stride time and calculated the intrinsic fractal frame that is hidden below the repetitive structure of physiological gait. To the best of our knowledge, this is the first study to analyse these characteristics in the Portuguese population.

Since there are no studies in Portugal, we discussed our results using information from other countries.

The study population was categorized using the CDC calculator. When we analysed the height and weight of the study participants, we found that only one of the five participants was of normal height and weight, with the rest being overweight and obese [2].

As expected from older adults, we observed in this study a lower fractal scaling as compared to what is typically observed in healthy young adults during overground walking [14, 15]. A mean value of α = 0.81 indicates that the older adults of the present study present fluctuations in their gait pattern that are more towards randomness (α = 0.5 is typically observed in white-noise type of signals). This represents a breakdown in the fractal structure (α ~ 1.0) of the gait patterns observed in healthy adults. This breakdown has previously been observed as an effect of aging and in the presence of neurological diseases [15].

Likewise, fallers older adults also exhibit a breakdown in fractal scaling compared to non-fallers [4]. Therefore, restoring the fractal structure in older adults can possibly result in other health-related outcomes.

This study provides evidence that a smartphone might provide valid measure to assess the spaciotemporal characteristics of gait and provide gait variability training.

## Data Availability

The datasets used and/or analysed during the current study are available from the corresponding author on reasonable request.

## Abbreviations

CDC: Centers for disease control and prevention
DFA: Detrended fluctuation analysis
ISIs: Inter-stride intervals
α-ISIs: ISIs time series
MA: Municipality of Almada
UE: University of Évora
